# Comparative Analysis of Periodontal Parameters and Patient Satisfaction Utilising Different Temporary Crown Fabrication Techniques: A Parallel-Group Randomised Controlled Trial

**DOI:** 10.7759/cureus.56977

**Published:** 2024-03-26

**Authors:** Amrutha Shenoy, Subhabrata Maiti, Deepak Nallaswamy

**Affiliations:** 1 Department of Prosthodontics, Saveetha Dental College and Hospitals, Saveetha Institute of Medical and Technical Sciences, Saveetha University, Chennai, IND

**Keywords:** health, quality of life (qol), periodontal health, patient satisfaction, pink esthetic score (pes), direct-indirect technique, temporary crowns

## Abstract

Aim

The study aims to evaluate the efficacy of different techniques for temporary crown fabrication in maintaining periodontal health and patient satisfaction, addressing a critical gap in the existing literature and informing evidence-based clinical practices.

Materials and methods

This study, conducted in accordance with CONSORT guidelines, was a parallel-group randomised trial conducted at a dental institute in India. In total, 36 participants aged 18-65 requiring anterior tooth region crowns were randomly assigned to three groups: direct (control), indirect (Test Group 1) and direct-indirect (Test Group 2) fabrication techniques. Participants were selected from outpatient departments based on eligibility criteria, and interventions were allocated using randomization tables. Outcome assessments included gingival health metrics and patient satisfaction levels, with statistical analyses performed using the Kruskal-Wallis test.

Results

Significant differences were observed in the pink esthetic score (PES) and patient satisfaction (P=0.029) among the three groups, with the direct-indirect technique group demonstrating the highest median PES (9 out of 10). However, no significant disparities were noted in the plaque index (PI) or probing depth (PD) among the groups.

Conclusion

The direct-indirect technique demonstrated superior PES and patient satisfaction, indicating potential benefits for periodontal health and patient experiences. Integration of virtual preparation workflows may optimise outcomes, but further research is needed for validation and guideline development.

## Introduction

Preserving periodontal health is fundamental to comprehensive oral care, with temporary crowns serving as crucial elements in maintaining dental integrity during restorative and prosthetic procedures until the patient receives their final prosthesis [1]. Traditional or direct methods for fabricating temporary crowns often involve meticulous manual waxing-up of diagnostic casts, recording a putty index of the mock-ups, followed by adding acrylic resin/bis-acryl composite temporary crown and bridge materials into the putty index and placing them on the prepared tooth surfaces, followed by intricate finishing and polishing procedures [2]. However, despite their widespread use, these conventional techniques are time-consuming and frequently yield suboptimal crown margins, potentially leading to post-procedural soft tissue complications around the tooth [3]. A notable concern arises from the observed occurrence of gingival inflammation or irritation near temporary crown margins, often detected during subsequent appointments [4]. This clinical observation underscores the need to scrutinise the effectiveness of conventional fabrication methodologies in achieving optimal periodontal outcomes [5].

The advent of digital dental technologies represents a transformative shift in temporary crown fabrication, offering enhanced precision and efficiency compared to traditional methods [6]. Among these advancements, 3D printing and milling technologies have emerged as prominent solutions with distinct capabilities [7, 8]. 3D printing, also known as additive manufacturing, involves the layer-by-layer deposition of materials to construct three-dimensional objects [9]. In contrast, milling utilises computer-controlled cutting tools to carve out temporary crowns from solid blocks of material. This subtractive manufacturing process allows for precise control over crown dimensions and surface finish, resulting in temporaries with exceptional accuracy and smooth margins [10]. Common materials used for milling include polymethyl methacrylate (PMMA) and composite resin blocks, known for their biocompatibility and durability [11]. Notably, milled temporary crowns often exhibit smoother surfaces and sharper margins compared to their 3D-printed counterparts [12]. Despite the advantages of 3D printing, milling holds distinct benefits, particularly in terms of surface quality and material properties [13]. The superior surface finish and wider range of material options afforded by milling make it an attractive choice for achieving optimal periodontal health outcomes and patient satisfaction [14].

Despite the promise of digital solutions [15], a conspicuous gap exists in the scientific literature regarding the comparative efficacy of different temporary crown fabrication techniques in influencing periodontal parameters and patient satisfaction [16]. Existing research tends to focus on isolated aspects of temporary crown construction, relegating comprehensive evaluations of their impact on periodontal health and patient experiences to the periphery [17, 18]. Consequently, a critical gap remains, hindering the formulation of evidence-based guidelines for optimal temporary crown fabrication practices in contemporary clinical settings. The present study aims to address the comparative efficacy of three distinct temporary crown fabrication techniques, namely direct (involving chair-side fabrication of temporary crowns), indirect (involving laboratory fabrication of crowns), and direct-indirect (involving a combination of direct and indirect methods) by assessing their impact on key periodontal health metrics, including gingival health indices, periodontal pocket depth, and attachment loss, as well as evaluating patient satisfaction levels with factors such as comfort, aesthetics, and durability. The null hypothesis was that there would be no significant difference among the techniques in terms of their impact on periodontal parameters and patient satisfaction outcomes.

## Materials and methods

Study design

This study adhered to the CONSORT guidelines [19] and was designed as a parallel-group randomised trial conducted at the Prosthodontics Department of Saveetha Dental College and Hospitals, Chennai, India. A total of 36 participants aged between 18 and 65 years, requiring crowns on the left central incisor (FDI annotation: 21), were selected through simple random sampling from the outpatient pool. Sample size calculation was based on data obtained from a previously published study [20], using an alpha level of 0.05, power of 0.80, and anticipated effect size. The calculated sample size was adjusted for potential dropouts. The study protocol obtained approval from the institutional review board's ethical clearance committee (Approval number: IHEC/SDC/PROSTHO-2002/23/021) and was registered with the Clinical Trials Registry, India (CTRI/2024/03/064511). Prior to initiating treatment, all patients were given a thorough explanation of the study's objectives and methodology. Additionally, written consent was obtained from each participant prior to their enrollment, ensuring adherence to the ethical principles outlined in the Declaration of Helsinki during the study's conduct.

Participants

Participants were recruited from the outpatient departments of endodontics and prosthodontics and included individuals requiring temporary crowns in tooth region 21 as part of restorative or prosthetic dental procedures. Participants eligible for inclusion in the study were those aged between 18 and 65 years, requiring temporary crowns specifically in the anterior tooth region (FDI annotation: 21), and willing to provide written informed consent. Additionally, they were required to have good oral hygiene (Loe and Silness Plaque Index ≤ 2). Exclusion criteria encompassed individuals with severe periodontal disease impacting tooth stability, pregnant or lactating individuals, those with systemic diseases potentially affecting oral health or treatment outcomes, individuals with a history of allergy or adverse reactions to PMMA used in temporary crown fabrication, untreated dental caries or active oral infections, extensive dental restorations or prosthetic work necessitating immediate full-mouth rehabilitation, and those with a history of non-compliance with dental treatment or follow-up appointments.

Randomization and blinding

Participants were randomly assigned to one of three temporary crown fabrication techniques - direct, indirect, or direct-indirect - using a freely available computer-generated randomization tool for generating random numbers or sequences (https://www.randomizer.org/). Allocation concealment was ensured through the sequentially numbered, opaque, sealed envelope (SNOSE) technique [21], where the randomization group was written on a piece of paper and kept in an opaque sealed envelope labelled with a serial number. Due to the nature of the interventions, blinding of participants and clinicians was not feasible. However, outcome assessors and data analysts were blinded to the assigned intervention groups to minimise bias.

Interventions

Direct Technique (Control Group)

The direct technique involved the chair-side fabrication of the temporary crown after tooth preparation. This process included meticulous manual waxing-up of diagnostic casts, recording a putty index of the mock-ups, and subsequently adding acrylic resin into the putty index. The materials were then carefully placed on the prepared tooth surfaces, followed by finishing and polishing procedures.

Indirect Technique (Test Group 1)

Temporary crowns were fabricated in the laboratory using a digital designing workflow (3Shape Dental Manager, Niels Juels Gade 13, 1059 Copenhagen, Denmark) wherein a single-stage putty light body impression (Zhermack®, Badia Polesine, Italy) of the prepared tooth was scanned using an extraoral scanner (E4 lab scanner, 3Shape Dental Manager). The designed file was later milled using PMMA in the in-house 5-axis milling machine (imes-icore, CORiTEC 350i milling machine®, Eiterfeld, Germany) to obtain the temporary crown.

Direct-Indirect Technique (Test Group 2)

**Figure 1:**
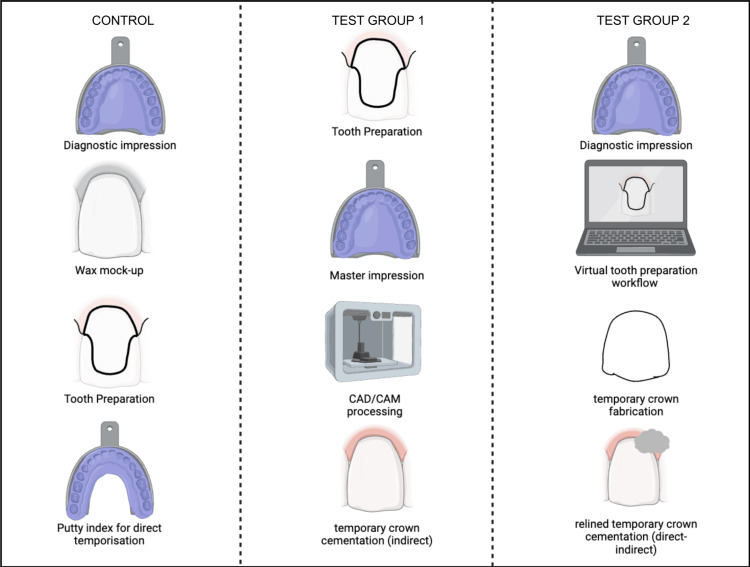
Workflow of each technique depicting the chair-side manual fabrication for Group 1 (direct technique), laboratory-based CAD/CAM for Group 2 (indirect technique), and digital fabrication with chair-side relining for Group 3 (direct-indirect technique). CAD/CAM: computer-aided design and computer-assisted manufacturing Image credit: Amrutha Shenoy

**Figure 2:**
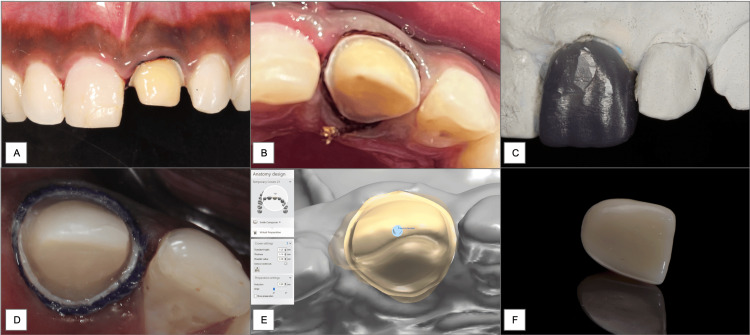
Tooth preparation (A), gingival retraction (B), wax mock-up of tooth to receive temporary crown using direct technique (C), prepared tooth for indirect technique (D), virtual tooth preparation workflow to obtain temporary crown using the direct-indirect method (E), temporary crown fabricated through direct-indirect technique (F).

Temporary crowns were digitally fabricated using a virtual tooth preparation workflow on the 3Shape software. Subsequently, these digitally fabricated temporary crowns were adjusted and relined chair-side with acrylic resin to ensure optimal fit and aesthetics, in accordance with the tooth preparation done by the clinician (Figures 1-2).

Outcomes

Primary Outcomes

The primary outcome measure focused on assessing various gingival health metrics and aesthetic outcomes. This included the pink esthetic score (PES), which evaluates gingival conditions on a scale of 0 to 10, with higher scores indicating better aesthetic outcomes [7]. The PES assesses five specific variables such as mesial papilla, distal papilla, curvature of the facial mucosa, level of the facial mucosa, and root convexity/soft tissue colour and texture at the facial aspect of the site, utilising a 2-1-0 score system. Furthermore, plaque accumulation was measured using the plaque index (PI), graded from 0 to 3, with higher scores indicating greater plaque accumulation. Before evaluating the PI, participants were instructed to brush their teeth using the modified Bass technique and were advised to brush their teeth twice daily. Probing depth (PD) was utilised to assess periodontal pocket depth, measured in millimetres, with deeper pockets indicating more severe periodontal disease. Lastly, bleeding on probing was evaluated as the presence or absence of bleeding upon probing, indicating gingival inflammation. These assessments were conducted during the subsequent appointment, one week after temporary crown placement.

Secondary Outcomes

This included evaluating patient satisfaction levels regarding the comfort, aesthetics, and durability of the temporary crowns using a structured questionnaire [22].

Statistical analysis

Outcome assessments were performed one-week post-temporary crown placement by an examiner (SM) blinded to the intervention allocation. To evaluate the differences among the three distinct temporary crown fabrication techniques (direct, indirect, and direct-indirect) regarding PES, PI and PD, the Kruskal-Wallis test was employed to determine if there were statistically significant differences among the three groups. This non-parametric test was chosen due to the non-normal distribution of the data. The significance level was set at p < 0.05. Adjustments for multiple comparisons were applied using appropriate methods, such as Bonferroni correction, to control the overall Type I error rate. Descriptive statistics, including median values and interquartile ranges (IQRs), were reported for each group. All statistical analyses were conducted using SPSS software v. 29.0 (IBM Corp., Armonk, NY).

## Results

**Figure 3:**
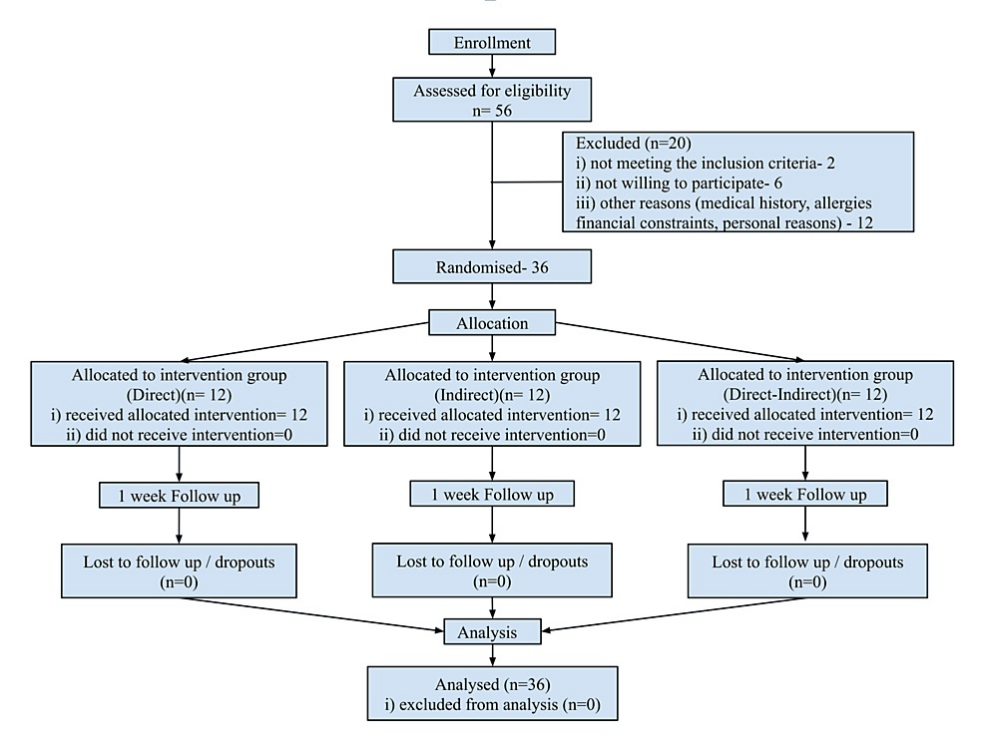
Flow diagram summarising participant enrollment, randomization, group assignment, intervention allocation, dropout details, and the final number of participants for analysis.

A total of 36 participants (25 males and 11 females) completed the study, and no adverse reactions to the materials used in any of the groups were reported. The flow diagram (Figure 3) offers a comprehensive summary of participant enrollment, randomization, group assignment, intervention allocation, and details regarding dropouts, culminating in the final number of participants included in the analysis.

**Figure 4:**
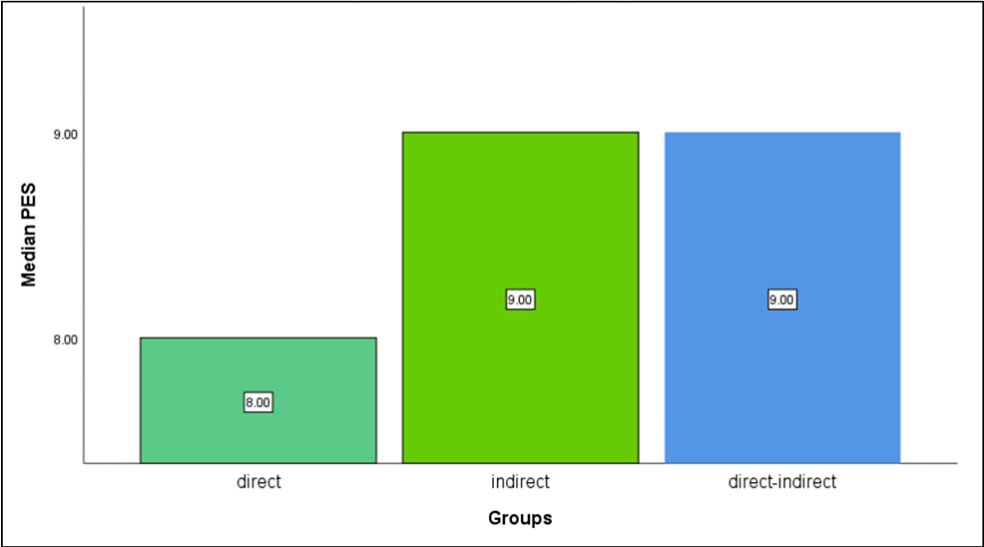
Difference in the median PES scores between the three groups at the one-week follow-up. Significant difference was seen between the groups (DI = I > D). The p-value was derived using a non-parametric Kruskal Wallis test. PES: pink esthetic score; DI: direct-indirect; I: indirect; D: direct

**Figure 5:**
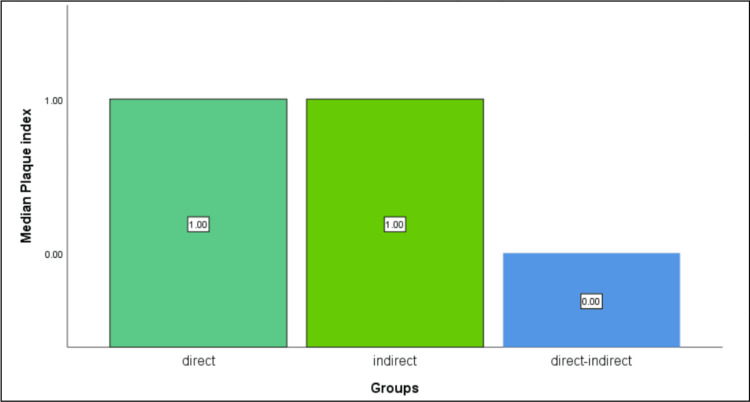
Difference in the median plaque scores recorded between the three groups at the one-week follow-up analysed using the Kruskal-Wallis test. No significant difference was found between the three groups.

**Figure 6:**
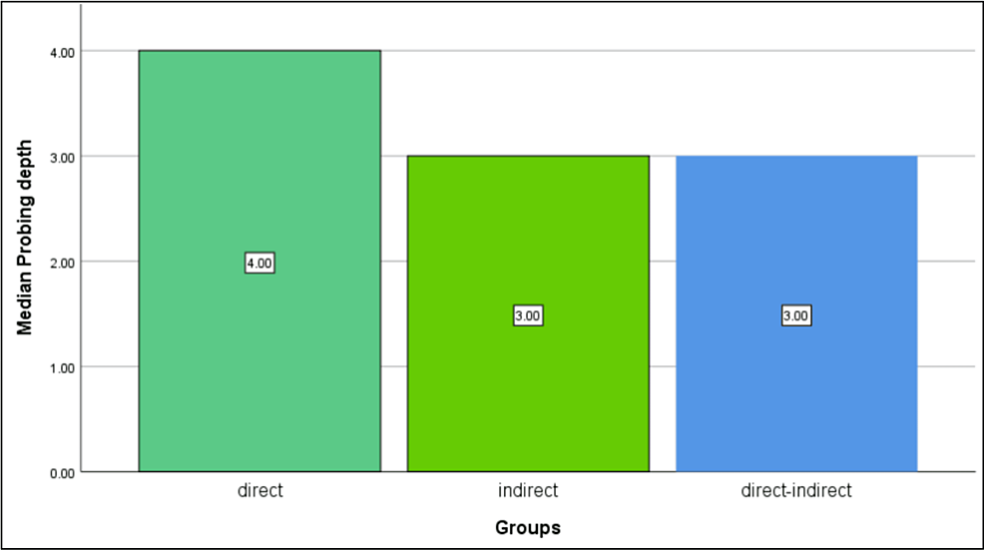
The difference in the median probing depth recorded between the three groups at the one-week follow-up was analysed using the Kruskal-Wallis test. No significant difference was found between the three groups.

The comparative analysis revealed significant differences in PES and patient satisfaction among the three groups (P=0.029). Notably, the direct technique group (control group) exhibited a median PES of 8 out of 10, while Test Group 2 (direct-indirect technique) demonstrated a higher median PES of 9 out of 10 (Table 1). Meanwhile, Test Group 1 (indirect technique) also displayed a median PES of 9 (Figure 4). Similarly, regarding patient satisfaction, the direct technique group reported moderate satisfaction levels, whereas Test Group 2 expressed notably higher satisfaction levels. Conversely, Test Group 1 reported standard levels of satisfaction. However, no significant differences (P>0.05) were observed in PI or PD among the three groups (Figures 5-6).

**Table 1:** Difference in the observed parameters for each at the one-week follow-up. Significant differences were observed in the PES score. *Significant at p<0.05, the p-value was derived using the non-parametric Kruskal Wallis test. PES: pink esthetic score

Outcome	Groups	N	Min. score	Max. score	Median	Mean rank	H value	P value
PES	Direct	12	7	8	8	4.20	7.075	0.029*
	Indirect	12	8	9	9	9.20		
	Direct-Indirect	12	8	9	9	10.60		
PI	Direct	12	0	2	1	10.00	2.216	0.330
	Indirect	12	0	1	1	9.20		
	Direct-Indirect	12	0	1	0	6.30		
PD	Direct	12	3	5	4	10.40	2.700	0.259
	Indirect	12	3	4	3	6.80		
	Direct-Indirect	12	3	4	3	6.80		

## Discussion

Preservation of periodontal health is crucial in comprehensive oral care, with temporary crowns playing a vital role in maintaining dental integrity during restorative and prosthetic procedures until the final prosthesis is received [23]. Traditional methods of temporary crown fabrication, often manual and meticulous, have been associated with time-consuming processes and suboptimal outcomes, potentially leading to post-procedural complications such as gingival inflammation or irritation [24]. This study aimed to compare the effectiveness of three distinct temporary crown fabrication techniques (direct, indirect, and direct-indirect) in achieving optimal periodontal outcomes and patient satisfaction levels. The results of the study revealed significant differences in PES and patient satisfaction among the three groups. The direct-indirect technique showed notably high PES scores and patient satisfaction levels compared to the direct and indirect techniques. However, no significant differences were observed in PI or PD among the groups. The lack of significant differences in PI or PD among the groups could be attributed to the short-term nature of the study, with the assessment conducted just one week after cementation.

The advent of digital dental technologies has revolutionised temporary crown fabrication, offering enhanced precision and efficiency compared to traditional methods [25]. 3D printing and milling technologies offer distinct advantages, with 3D printing providing versatility and rapid fabrication, while milling ensures precise control over dimensions and surface finish. Despite the benefits of 3D printing, milling stands out for its superior surface quality and material properties [26]. However, existing literature lacks comprehensive evaluations of the impact of different temporary crown fabrication techniques on periodontal health and patient experiences [27]. Most studies focus on isolated aspects of temporary crown construction, leaving a critical gap in evidence-based guidelines for optimal fabrication practices [28]. Moreover, considering the varying cytotoxicity of different provisional materials highlighted in a previously published study [29], clinicians should tailor their treatment plans to minimise adverse effects on gingival fibroblasts, ultimately improving the esthetic and biological success of the restorations. Similarly, the virtual preparation workflow has emerged as a transformative tool, allowing clinicians to digitally design and visualise dental restorations before any physical intervention [30]. Integrating this innovative technique into the direct-indirect temporary crown fabrication method offers several added benefits, serving as a valuable guide for both clinicians and patients. By enabling patients to visualise their proposed crowns before treatment initiation, it enhances patient understanding and engagement in the treatment process. This visual aid helps set realistic expectations and fosters a sense of confidence and trust between the patient and the dental team. Moreover, the direct-indirect technique offers advantages over traditional methods in terms of reducing soft tissue inflammation. With the precise digital design and fabrication of temporary crowns, adjustments during the chair-side relining process can be minimal and less time-consuming, resulting in decreased manipulation of the surrounding soft tissues. This minimally invasive approach contributes to reduced post-procedural inflammation and discomfort, thereby enhancing the overall patient experience.

The findings of the current study corroborate the benefits of the direct-indirect technique, demonstrating higher patient satisfaction levels and surrounding soft tissue outcomes compared to other fabrication methods. The combination of the virtual preparation workflow and chair-side relining not only ensures optimal fit and aesthetics but also reduces appointment time and reinforces patient satisfaction by involving them in the treatment decision-making process and minimising post-treatment complications [31].

However, despite the strengths of this study, several limitations need to be addressed. These include a short follow-up period and a lack of long-term data on the durability of temporary crowns. Additionally, the study was conducted at a single institution, limiting its generalizability to broader populations. Future research should focus on larger sample sizes and multicenter studies to validate the findings of this study. Furthermore, investigations into the cost-effectiveness of different temporary crown fabrication techniques would provide valuable insights for clinical practice. Overall, this study contributes to the growing body of evidence on temporary crown fabrication techniques and their impact on periodontal health and patient satisfaction, highlighting the potential benefits of integrating virtual preparation workflows into contemporary restorative dentistry practices.

## Conclusions

Based on the results obtained, the direct-indirect technique demonstrated notable advantages over traditional direct and indirect methods in terms of achieving higher PES and patient satisfaction levels. This indicates its potential to improve periodontal health outcomes and enhance patient experiences during temporary crown fabrication. While no significant differences were observed in PI or PD among the three groups, the direct-indirect technique's ability to minimise soft tissue inflammation highlights its clinical relevance. These findings underscore the importance of integrating virtual preparation workflows into contemporary restorative dentistry practices to optimise patient outcomes and minimise post-procedural complications. Further research with larger sample sizes and longer follow-up periods is warranted to validate these findings and inform evidence-based clinical guidelines.
